# American Medical Temperance Association

**Published:** 1903-04

**Authors:** 


### AMERICAN MEDICAL TEMPERANCE ASSOCIATION.

This Association was organized at Washington, D. C., May, 1891, at the meeting of the American Medical Association. Its members are very largely composed of members of the American Medical Association. Its first president, Dr. N. S. Davis of Chicago, so well known to all the profession in this country, has been continually re-elected to this office up to two years ago, when he was made Honorary president. The following is an extract from the principles of the Association:

"The object of this Association is to encourage and promote the clinical, therapeutical, pharmacological, and chemical study of alcohol in health and disease. It also aims to gather, compile, and make available the studies and experiments of medical men in all parts of the country concerning the use of alcohol, and to formulate such definite facts as can be utilized and made available in the practice of medicine. It is assumed that all physicians interested in the study of alcohol should approach it from the scientific side alone, unbiased by any personal consideration of custom or habit, and with no object other than to ascertain the facts concerning this question, irrespective of all possible conclusoins. This is the spirit and purpose of the Association, and it is entirely independent of any other object except the purely scientific question of the nature and character of alcohol.

"All regular practitioners of medicine may become members by a two-thirds vote of the Association at any regular meeting, after signing the following form of application and transmitting the same to the Secretary of the Association."

The twelfth annual meeting will be held at New Orleans, May 7th, during the session of the American Medical Association. A number of very important papers will be read on this subject, and the medical public are very cordially invited to attend. The following list of officers will give the reader some idea of the character of the Associaton: Honorary President, N. S. Davis, A. M., LL. D., Chicago, Ill.; President, W. S. Hall, Ph D., M. D., LL. D., Chicago, Ill.; Vice Presidents, H. O. Marcy, M. D., LL. D., Boston, Mass.; H. D. Didama, M. D., LL. D., Syracuse, N. Y.; T. A. MacNichol, Ph. D., M. D., New York City; Secretary, T. D. Crothers, M. D., Hartfort, Conn.; Corresponding Secretary, C. S. Stewart, M. D., Battle Creek, Mich.; Treasurer, G. W. Webster, M. D., Chicago, Ill. It will be interesting to know that seven of these officers are teachers and authors in the largest colleges in the country. The secretary will be very glad to communicate with any persons interested in this great subject. Address T. D. Crothers, M. D., Hartford, Conn.

